# Implementation fidelity of a pharmacist-led intervention program to improve a high serum phosphate concentration in haemodialysis patients: a mixed-methods study

**DOI:** 10.1007/s11096-025-01995-z

**Published:** 2025-09-26

**Authors:** F. J. van den Oever, E. C. Vasbinder, Y. C. Schrama, T. van Gelder, P. M. L. A. van den Bemt, J. G. Hugtenburg

**Affiliations:** 1 Department of Pharmacy, Franciscus Gasthuis and Vlietland, Kleiweg 500, 3045 PM Rotterdam, The Netherlands; 2 Department of Clinical Pharmacy and Toxicology, Leiden University Medical Centre, Albinodreef 2, 2333 ZG Leiden, The Netherlands; 3 Department of Clinical Pharmacy and Pharmacology, University Medical Centre Groningen, Hanzeplein 1, 9713 GZ Groningen, The Netherlands; 4 Department of Clinical Pharmacology and Pharmacy, Amsterdam University Medical Centre, De Boelelaan 1118, 1081 HZ Amsterdam, The Netherlands

**Keywords:** Adherence, Haemodialysis patients, Implementation fidelity, Process evaluation, The Netherlands

## Abstract

**Introduction:**

The PIDO-P (Pharmacist Intervention and Dose Optimization of Phosphate-binding medication) was designed to improve high serum phosphate concentration (SPC) in haemodialysis patients with a high pill burden of phosphate-binding medication (PBM). This intervention consisted of three pharmacist-patient consultations, in which barriers to adherence to PBM were addressed and PBM dose was reduced. Although this intervention improved PBM adherence, SPC remained high.

**Aim:**

To determine the implementation fidelity (IF) of the PIDO-P intervention.

**Method:**

This mixed-methods implementation study had a convergent design using the intervention mixed-methods framework. Data from all patients included in the PIDO-P study (n = 75) were used to assess IF using Carroll’s Framework for IF. Six key components were identified [A: identifying barriers, B: assessing medication-related health literacy; C: providing information and advice, D: discussing patient preferences, E: providing a summary/dose reduction advice, F: performing a follow-up consultation]. Two researchers independently rated the extent to which the different aspects of the key intervention components were carried out as planned. Data sources were research administration (quantitative and qualitative), oral surveys from patients (quantitative and qualitative) and pharmacists (quantitative), semi-structured interviews with six patients, two pharmacists, and three prescribers (qualitative), and electronic medical records (quantitative). Data from semi-structured interviews were thematically analysed according to Braun and Clarke with Atlas.ti, quantitative data were analysed using descriptive statistics in SPSS. Where possible, data integration was performed.

**Results:**

The adherence to the intervention was moderate to high, except for the screening process. The written summary was delivered to a moderate degree (65.3%). Facilitation strategies were helpful, and pharmacists considered the intervention not too complex. The quality of delivery and participant responsiveness were good. Four IF themes could be identified: (1) patient knowledge and understanding, (2) correct use of PBM, (3) PBM treatment individualisation, (4) relationship between pharmacist and patient. To increase its feasibility, the intervention should be targeted at patients with SPC > 2.0 mmol/L, and patient selection should be improved.

**Conclusion:**

The lack of effect of the PIDO-P intervention on SPC cannot be explained by low IF. Targeting patients with higher SPC and improving patient selection may increase its effectiveness.

**Supplementary Information:**

The online version contains supplementary material available at 10.1007/s11096-025-01995-z.

## Impact statements

- As implementation fidelity was high and there were no important points of improvement regarding the moderating factors, the PIDO-P intervention seems feasible in clinical practice, with a high willingness to participate in healthcare professionals and patients.
- The patient screening and selection process should be optimised to increase feasibility.
- To increase the effectiveness of the intervention, the intervention should target patients with higher serum phosphate concentrations.

## Introduction

Chronic kidney disease (CKD) is frequently complicated by a high serum phosphate concentration (SPC), which is difficult to manage. The proportion of haemodialysis patients with an SPC outside the target range may be as high as 55% [1]. Important consequences of a high SPC are cardiovascular morbidity and mortality [2]. Treatment of a high SPC in dialysis patients consists of three cornerstones: dietary phosphate reduction, phosphate removal during haemodialysis sessions, and treatment with phosphate-binding medication (PBM) [3].

Suboptimal adherence occurs in approximately 50% of the patients using PBM [4] and is associated with increased morbidity and mortality and higher healthcare costs [5]. Suboptimal PBM adherence often results in a high SPC, unresponsive to treatment with PBM. This unresponsiveness frequently provokes a negative treatment spiral, in which the nephrologist keeps increasing the dose, leading to an even lower PBM adherence [6].

Several educational and behavioural interventions, including pharmacist interventions, have been studied to improve suboptimal adherence to PBM [7-13]. These interventions mostly demonstrated short-term positive effects on SPC [14], knowledge, and adherence to PBM [7, 9–11, 14]. Multi-component interventions, combining educational and behavioural aspects, tailored to the problems and challenges of the individual patient, seem most promising in improving adherence [5, 14].

The PIDO-P (Pharmacist Intervention and Dose Optimization of Phosphate-binding medication) study investigated a multi-component pharmacist intervention consisting of pharmacist-patient consultations, incorporating a dose reduction of PBM, in patients on haemodialysis with a high SPC and a high PBM pill burden (see Box 1).

**Box 1:**
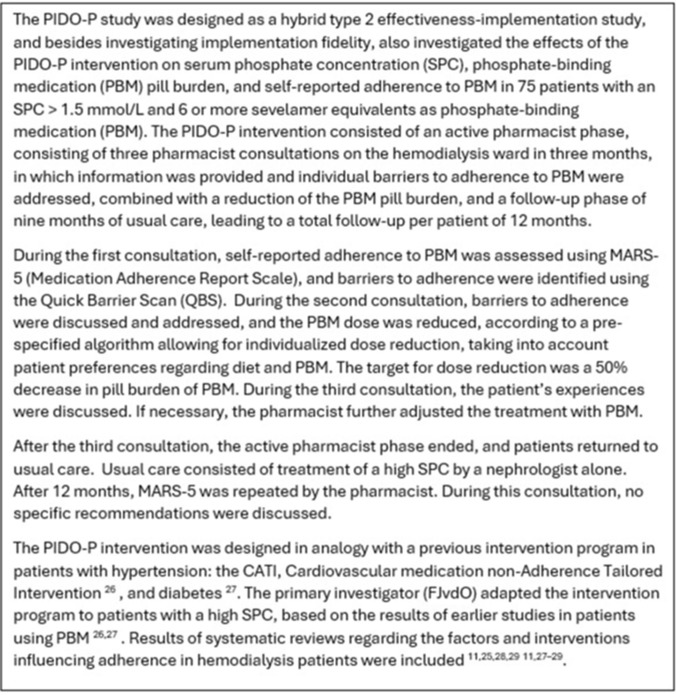
Methodology of the pharmacist intervention and dose optimisation of phosphate-binding medication (PIDO-P) study

The PIDO-P intervention improved self-reported adherence to PBM and reduced PBM pill burden, but, contrary to our expectations, did not reduce SPC after three months. To interpret these findings, it is important to understand how the intervention was implemented and performed, as it is known that the effectiveness of an intervention is related to the intervention being delivered as intended [15].

### Aim

This study aimed to evaluate the implementation fidelity (IF) of the PIDO-P intervention, including potential moderating factors that might have influenced the implementation and performance of this intervention. Furthermore, the feasibility of implementation in clinical practice, including its barriers and facilitators, was explored.

## Method

### Study design

**Fig. 1:**
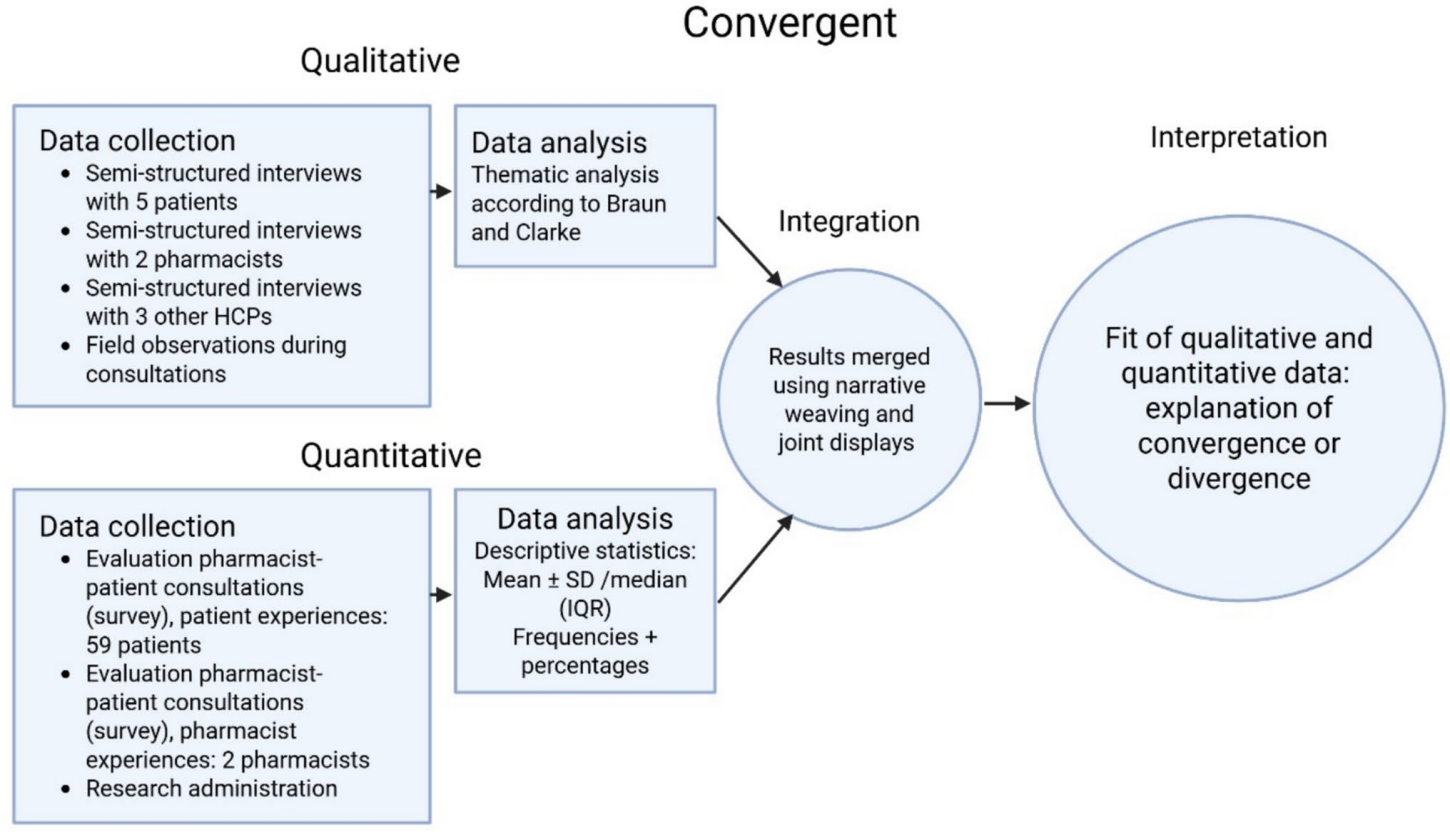
Procedural diagram

This study evaluated IF with Carroll’s Framework for IF (CFIF, see below), and had a mixed-methods design in analogy with earlier studies on the implementation of pharmacist intervention [16, 17]. This IF study was part of the PIDO-P study, an effectiveness-implementation hybrid type 2 study (see Box 1), and had a convergent design using the intervention mixed-methods framework [18, 19]. Figure 1 depicts the procedural diagram.

Integration was performed by connecting, building, and merging [18, 19]. Methods were integrated by connecting, as the patient sample for the semi-structured interviews was selected from the subset of patients without signs of limited medication-related health literacy. Building was performed using data from the oral patient surveys to design the topic lists for the semi-structured interviews with patients. Merging was performed at the interpretation and reporting level using joint displays and a narrative weaving approach. If applicable, metainferences were generated.

### Study population

Data from all patients included in the PIDO-P study (n = 75) were used to evaluate IF. At baseline, patients were around 65 years of age (65 ± 16, mean ± SD); 65% were male; patients used 12.7 ± 5.0 different medications with a total pill burden of 22.3 ± 7.7. PBM pill burden was 8.8 ± 2.9. Their main renal diagnoses were hypertension (30.7%) and diabetes mellitus (20.0%).

### Conceptual framework for implementation fidelity

**Fig. 2:**
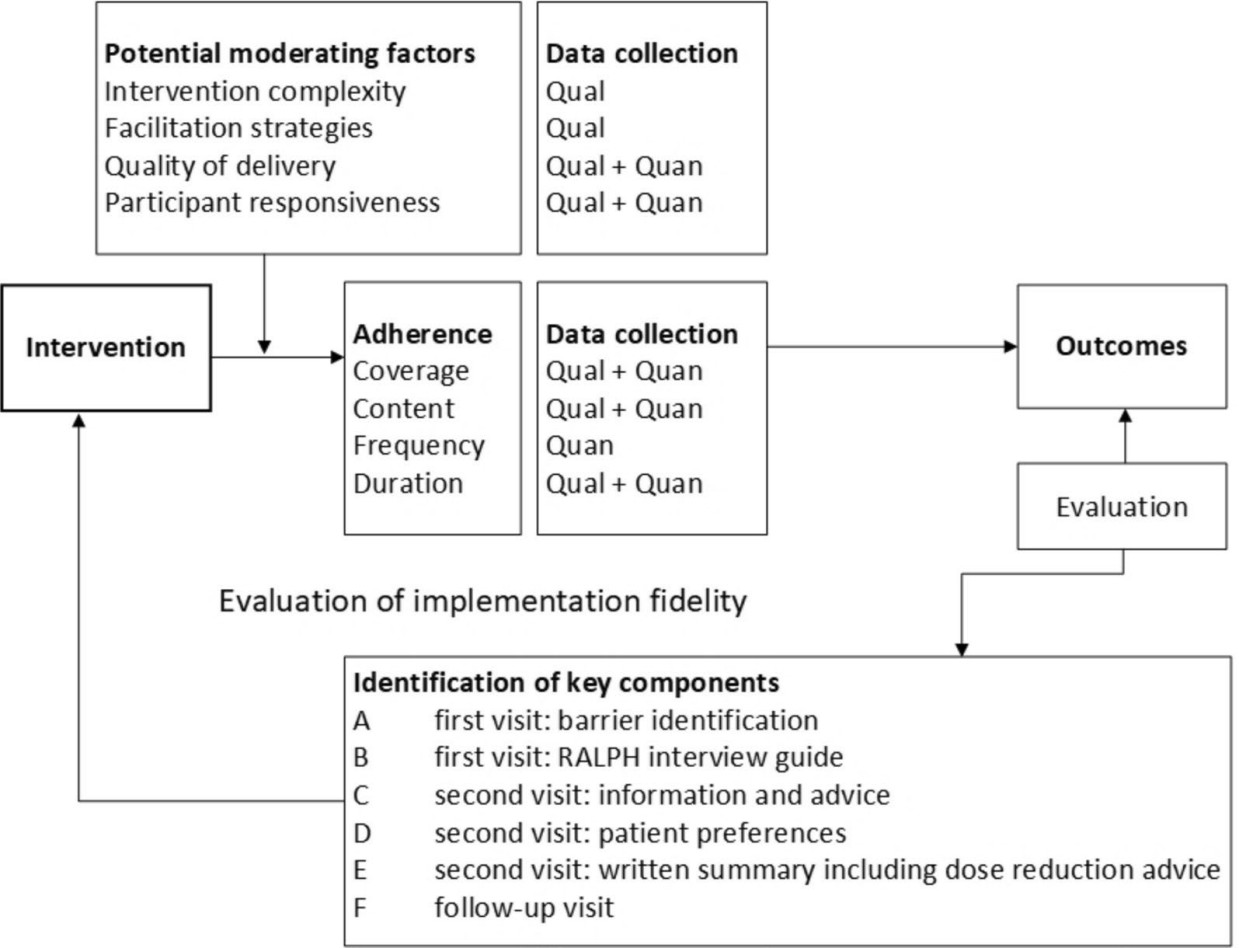
Carroll’s conceptual framework for implementation fidelity (CFIF)

We evaluated the IF of the intervention using Carroll’s CFIF [20]. This framework consists of two interrelated components: adherence to the intervention and moderating factors. Figure 2 shows the components of CFIF, including their mutual relationship, and their relationship with the outcomes of the intervention.

#### Adherence to the intervention

Adherence to the intervention can be defined as “the intervention being delivered as designed” [20]. Adherence includes the following components: *coverage* (the degree to which patients who should receive the intervention, actually do so), *content* (the degree to which all key intervention components are implemented), *frequency* (the degree to how often the elements of the intervention are delivered) and *duration* (the degree to how long the intervention elements are delivered). *Frequency and duration* together can also be defined as dosage or exposure [20], or the amount of the intervention that participants have received. The adherence to the intervention was assessed by evaluating all key intervention components, which are listed below.

##### Key intervention components

Before the start of the PIDO-P study, the following six key intervention components were identified:

(A) First consultation: identification of barriers to PBM adherence using the Quick Barrier Scan (QBS)
(B) First consultation: RALPH (Recognising and Addressing Limited PHarmaceutical literacy) interview guide to explore medication-related health literacy
(C) Second consultation: information and advice, according to the selected modules from the TIG (Tailored Intervention Guide)
(D) Second consultation: patient preferences regarding PBM
(E) Second consultation: handwritten summary including dose reduction advice. We chose to provide a handwritten summary for two reasons: (1) to be able to provide it directly after the consultation, and (2) a high percentage of dialysis patients do not use electronic devices and email
(F) Follow-up consultation after three months, in which patient experiences were discussed and the intervention was evaluated

For each key intervention component and each of the adherence components (*coverage, content, frequency, and duration*), specific research questions were defined, and based on these questions, the IF was rated (see next section). These research questions are described in Table 1 and were based on earlier IF research of pharmacist interventions using CFIF [16, 17, 21].

**Table 1:** Overview of adherence to the intervention

Adherence	Research questions	Qualitative/quantitative data	Data source	Outcomes (including quotes from qualitative data, if applicable)	Metainferences	Rating
*Coverage*						Mode-rate
In general	How was the selection of patients perceived by pharmacists?	Qual/quan	I	Pharmacists: Inefficient and labour-intensive screening and selection process Repeated screening necessary, even in patients with stable exclusion criteria (for example, residing in a nursing home, or insufficient language proficiency)	Data were concordant. The inefficiency of the screening process lowered the effectiveness of the intervention and is very likely to hamper future implementation in clinical practice. A possible solution could be automatically combining medication data (type of PBM, number of PBM tablets) and SPC	Low
	Was the selection procedure performed according to the study protocol?	Qual	I	The patients were included according to the inclusion and exclusion criteria defined in the study protocol		High
	What proportion of patients was eligible? And what were the reasons for patients not being eligible?	Quan	I, IV	21.7% of screened patients were eligible n = 27 (74.0%) excluded for not meeting inclusion criteria n = 16 (4.3%) excluded for other reasons n = 5 (1.4%) denied participation		Low
	What proportion of eligible patients were invited to participate?	Quan	I, IV	All eligible patients (n = 80) were invited to participate	n.a	High
	What proportion of invited patients participated?	Quan	I, IV	75 of 80 (93.7%) invited patients participated	n.a	High
	What proportion of invited patients declined participation? And what were the reasons?	Quan	I	5 of 80 patients (6.3%) declined participation, most often due to no interest	n.a	High
	What reasons did patients provide for participation?	Qual/quan	I, III–VI	26 patients were asked to provide a reason for participation in the oral surveys (open-ended question), n = 9 (34.6%): receiving more information n = 3 (11.5%): PBM dosage reduction n = 3 (11.5%): contribution to science n = 3 (11.5%): distraction during dialysis n = 8 (30.8%) no specific reason In general, patients were interested and very willing to participate in the study when being told about the intervention and the reduction in pill burden Ph 1:”*They [the patients] say: We are here during dialysis and somebody from the pharmacists comes and visits us. Our visit was a welcome distraction and provided a possibility to chat with someone. I did not hear about the goal to lower phosphate from patients*.” Ph 2: “*Patients appreciated somebody visiting them and asking about them and their medication use. But they sometimes missed the relevance*.”	Data from different sources were discordant. A substantial part of the patients did not provide a reason for participation. The reduction in pill burden appealed to patients and was probably the most important reason for participation. Receiving information and increasing knowledge, were seen as positive “side effects” by patients, whereas for pharmacists and prescribers the attention to SPC and PBM were the most important aspects of the intervention	Low
	How was the drop-out rate during the 3-months follow-up and the 12-month follow-up?	Quan	I	Follow-up at 3M: Complete n = 72 Transplantation n = 2 Not able to continue consultations n = 1 Follow-up at 12M: Complete n = 54 Transplantation n = 10 Death: n = 8 Transfer to another hospital n = 2 Not able to continue consultations n = 1	n.a	High (at 3M); Moderate (at 12M)
*Content*						
A. 1 st consultation—barrier identification B. 1 st consultation – RALPH	To what extent were the different components of the 1 st consultation delivered as planned?	Qual/quan	I, V, VI	Key components A and B performed in all patients Patients and pharmacists reported a lack of knowledge about SPC and PBM Barriers to adherence: Lack of knowledge/information n = 50 (66.6%) Negative beliefs n = 33 (44.0%) Forgetfulness n = 32 (42.6%) Side effects n = 19 (25.3%) Medication-related health literacy [22, 23] ≥ 1 difficulty in total n = 61 (81%) ≥ 1 difficulty in the critical domain n = 59 (79%) Indication unknown n = 29 (39%) Patient does not search information n = 36 (48%) Problems with shared decision-making n = 25 (33.3%) Ph 1: “*They don’t know how it works. They heard it from me.*” Ph 2: “*They don’t understand some things. They just take it and that’s it*.”	Data were concordant. Patients and pharmacists both reported a lack of knowledge regarding PBM as barrier to adherence. A substantial part of the patients seemed subdued regarding this lack of knowledge Limited health literacy occurs frequently in this patient population and probably contributes to suboptimal adherence and low patient engagement Searching for ways to promote low-level patient participation may improve shared decision-making	High
C. 2nd consultation—information & advice D. 2nd consultation—patient preferences E. 2nd consultation—written summary including dose reduction advice	To what extent were the different components of the 2nd consultation delivered as planned?	Qual/quan	I–VI	The pharmacist provided Information and advice and discussed patient preferences n = 73 (97.3%) A written summary n = 49 (65.3%) Dose reduction advice n = 73 (97.3%) PBM dosage was reduced in 71 patients (94.7%) After 3M, n = 60 (80.0%) still used a lower PBM dosage Reduction in PBM pill burden at 3M was 3.1 ± 2.6 sevelamer tablet equivalents (n = 72). Patients reported improved knowledge (34/39 patients, 87.1%). Pharmacists reported improved understanding in 30/38 patients (78.9%). Both pharmacists and patients perceived the usefulness of the summary as limited. Pharmacists struggled with patient passivity. Ph 1: “*The biggest problem for me is that the patient is very passive*.” Ph 2: “*The partners search information; the patients are not concerned with that.*” Pr 1: “*Most of them really appreciated being able to lower the number of tablets*. “	Data were concordant The current information strategy regarding SPC and PBM by HCPs lacks effectiveness and does not meet the needs of most haemodialysis patients Patients do not feel a high SPC, nor do they feel the difference between being adherent or not being adherent to PBM. Therefore, most patients do not feel the urgency to improve PBM use The dose reduction appealed to patients. Therefore, it might be an interesting strategy to start a conversation about PBM, engage patients in self-management, and improve adherence to PBM	Moderate (written summary)—high for other compo-nents
F. Follow-up (3rd) consultation	To what degree was the follow-up consultation delivered as intended?	Qual/quan	I, III–VI	The follow-up (3rd) consultation was delivered as intended in 72 patients (96%). 52/59 patients (88.1%) reported the consultations helped them to correctly use PBM. Pr 1: “*The study’s attention helps to focus on the tablets and the importance of taking them*.” Pr 2: “*The attention is good*.” Pr 3: “*Motivational interview techniques and counselling will definitely lead to patients better taking them, I am sure of that*.”	Data were concordant. Patients, pharmacists, and prescribers considered the intervention beneficial	High
*Frequency and duration*						
A. 1 st consultation—barrier identification B. 1 st consultation – RALPH	What was the number of 1 st consultations? Where key components A and B performed in all patients?	Quan	I	A 1 st consultation was conducted for all 75 patients. In all patients, key components A and B were performed	n.a	High
C. 2nd consultation—information & advice D. 2nd consultation—patient preferences E. 2nd consultation—written summary including dose reduction advice	What was the total number of second consultations? Where key components C to E performed in all patients?	Quan	I	A 2nd consultation was conducted for 73 patients (97.3%). In all 73 patients, key components C and D were performed. Regarding key component E, dose reduction advice was provided in all patients, whereas a written summary was provided in 65.3% of the patients	n.a	High
F. Follow-up (3rd) consultation	What was the total number of follow-up consultations?	Quan	I	A follow-up (3rd) consultation after 3M was conducted for 72 patients (96.0%)	n.a	High
*General*						
	What was the average consultation time?	Quan	I	The median consultation time for the 1 st consultation was 25 min (IQR 20–35 min). For the 2nd consultation, the mean duration was 21.5 ± 10.5, and for the 3rd consultation 14.4 ± 5.5 min	n.a	High
	What was the average number of days between the consultations?	Quan	I	The median number of days between the 1 st and 2nd consultation was 14 days (IQR 12–14). The median number of days between the 2nd and 3rd consultation was 91 days (IQR 84–97.5)	n.a	Not scored

I: study administration by the researchers; II: pharmacist perspective on pharmacist-patient consultations, oral survey; III: patient perspective on pharmacist-patient consultation, oral survey; IV: semi-structured interviews with pharmacists; V: semi-structured interviews with patients; VI: semi-structured interviews with prescribers; VII: electronic medical records

HCPs, healthcare professionals; IQR, interquartile range; N.a., not applicable; PBM, phosphate-binding medication; Ph, pharmacist; Pr, presciber; RALPH, recognising and addressing limited pharmaceutical literacy; SPC, serum phosphate concentration; TIG, tailored intervention guide

##### Rating the adherence to the intervention

Two researchers (FJvdO and JGH) independently rated the extent to which the different aspects of the key intervention components were carried out as planned (i.e. whether the adherence to the intervention for this key component was low, moderate, or high). “Low” was defined as the key intervention component being performed in less than 50% of the patients, “moderate” as in 50% to 75% of the patients, and “high” as in more than 75% of the patients.

### Moderating factors

Moderating factors include intervention complexity (to which degree the complexity of the intervention is an important barrier to its adoption), facilitation strategies (the degree to which strategies, such as training and intervention materials are delivered), quality of delivery (is the intervention delivered in a way appropriate to achieving what was intended), and participant responsiveness (to what extent patients and pharmacists are committed to the intervention). Based on earlier research on pharmacist interventions using CFIF [16, 17, 21], research questions regarding the moderating factors were formulated (see Table 2).

**Table 2:** Moderating factors and feasibility

Moderating factors	Research questions	Qualitative/quantitative data	Data source	Outcomes (including quotes from qualitative data, if applicable)	Metainferences
*Intervention complexity*					
A. 1 st consultation—barrier identification B. 1 st consultation – RALPH	How detailed was the protocol description of the first consultation?	Qual	I, IV	The pharmacists thought the protocol description of the first consultation was generally clear and informative. Ph 1: “*The protocol was clear.*”	N.a
	How complex were the different components of the first consultation?	Qual	IV	The pharmacists positively evaluated the intervention materials (QBS, RALPH, TIG). Ph 2: “*In terms of questionnaires, it was clear.*”	N.a
C. 2nd consultation—information & advice D. 2nd consultation—patient preferences E. 2nd consultation—written summary including dose reduction advice	How detailed was the protocol description of the second consultation?	Qual	IV	With the protocol and the intervention materials the pharmacists were able to discuss the selected intervention modules (IMs) from the TIG effectively	N.a
	How complex were the different components of the second consultation?	Qual	IV	The pharmacists stated that the second consultation did not contain complex aspects, but reported it was logistically challenging to provide a written summary. Furthermore, the patients generally did not use this summary. Ph 2: “*That [summary] was not read, we provided it, it went home and that was it. Even if they could read, they were not into that*.”	N.a
F. Follow-up consultation	How detailed was the protocol description of the follow-up consultation?	Qual	IV	During the evaluation, pharmacists stated that the follow-up consultation needed some explanation. Therefore, pharmacists received extra information and training regarding this consultation. Ph 1:”*There were some things in the protocol that did not match the implementation in practice, though.*”	N.a
	How complex was the follow-up consultation?	Qual	IV	The pharmacists stated that the follow-up consultation did not contain complex aspects. Ph 2: “*So it was not that complicated*.”	N.a
*Facilitation strategies*					
A. 1 st consultation—barrier identification B. 1 st consultation – RALPH C. 2nd consultation—information & advice D. 2nd consultation—patient preferences E. 2nd consultation—written summary including dose reduction advice F. Follow-up consultation	What strategies were used to facilitate the implementation of the consultation?	Qual	I, IV, VI	The pharmacists received instructions regarding the study protocol and study materials. Furthermore, all pharmacists were individually trained on topics of phosphate, phosphate-binding medication, adherence, and motivational interviewing. Consultation skills were individually trained with decreasing levels of supervision over time. Ph 1: “*She joined the patient consultations in the beginning, so I could observe her performing the consultations*”	N.a
	How did pharmacists perceive these strategies?	Qual	IV	The pharmacists described the training and support they received as sufficient and beneficial, in practical applicability as well as in content. Ph 2: “*I didn't really miss things, in terms of support in the consultations*.”	N.a
	How did prescribers perceive these strategies?	Qual	VI	Prescribers felt well informed about the study and its aim. However, they struggled to keep track of patients actively participating in the study, in whom they were not meant to change the PBM dose (during the first three months of follow-up). Pr 1: “*In the beginning, it was not entirely clear to me when someone had finished the study.*”	N.a
*Intervention quality of delivery*					
A. 1 st consultation—barrier identification B. 1 st consultation – RALPH	How did the pharmacists assess the quality of the different elements of the first consultation?	Qual/quant	II, IV	Evaluation of consultation 1 was performed for 43 patients. The pharmacists were generally positive about the quality of delivery. They were positive about patients’ understanding of the questions (90.7%)	The pharmacist-patient consultations improved patient knowledge and understanding about PBM. The pharmacist-patient consultations also helped patients solve practical problems with PBM and improved the correct use of PBM. Furthermore, the consultations improved shared decision-making and PBM treatment individualisation. The consultations helped building the relationship between pharmacist and patient See further the Themes in the joint display in Table 3
	How did the patients assess the quality of the different elements of the first consultation?	Qual/quant	III, V	The quality of the 1 st consultation was evaluated in 43 patients. All patients felt listened to by the pharmacist. They reported that they appreciated the consultations and were satisfied with the information. Their preferences regarding PBM were discussed during the consultation. 93.0% of the patients reported they understood all the questions	
C. 2nd consultation—information & advice D. 2nd consultation—patient preferences E. 2nd consultation—written summary including dose reduction advice	How did the pharmacists assess the quality of the different elements of the second consultation?		II, IV	Evaluation of consultation 2 by the pharmacist was performed in 38 patients. The pharmacists were generally positive about the quality of delivery. The pharmacists reported that providing a written summary was impractical and that patients often did not use this summary	
	How did the patients assess the quality of the different elements of the second consultation?	Qual/quan	III, V	Patient evaluation of consultation 2 was performed in 36 patients. Patients felt involved with shared decision-making regarding dose adjustments and choice for a specific PBM. All patients felt listened to by the pharmacist and felt free to ask questions	
F. Follow-up (third) consultation	How did the patients assess the quality during the follow-up (third) consultation?	Qual/quan	III, V	The quality of the follow-up consultation was assessed in combination with the consultations 1 and 2 (see row below)	
General		Qual/quan	III, V	In 59 patients, the first three consultations were evaluated. Patients were generally satisfied with the consultations. They felt that the information and advice they received helped them to take the medication correctly. 84.7% of the patients thought that the number of consultations was sufficient. 77.2% of the patients would recommend the intervention to other patients	
*Participant responsiveness*					
General	How engaged and satisfied were participants (patients and pharmacists) with the intervention?	Qual/quan	II, III, IV, V	The patients were mostly positive about the intervention: the score they provided was 8.2 ± 0.9 (mean ± SD). Pt 1:”*The consultations were interesting*.” Patients appreciated the attention and information The pharmacists felt engaged but struggled with patients’ passive attitude: Ph 1: “T*he biggest problem for me is that the patient is very passive.*” Ph 2: “*At times, it seemed like I really had to convince them*.” Patients and pharmacists believed that a single pharmacist conducting all consultations for one patient would be beneficial. Ph 1: “*The patients have to trust you”*	All involved parties felt engaged and were willing to participate, although their motivation was different. For patients, dose reduction and personal attention were important, whereas pharmacists valued the consultations and their role in guiding patients using PBM. Prescribers valued the recurrent attention to phosphate and PBM and the intensive collaboration and communication with the pharmacists All involved parties felt satisfied, although the passiveness of patients and their lack of interest in this topic sometimes decreased the motivation of pharmacists and prescribers in guiding patients using PBM. This passiveness has been described earlier in patients with low health literacy (HL)[27]. In the PIDO-P study, > 80% of patients using PBM had difficulties with medication-related HL [22]. Therefore, communication and information strategies should meet the needs of patients with limited HL. Using graphical information, applying the teach-back method, and limiting the amount of information are important strategies for patients with limited HL
	How engaged and satisfied were prescribers with the intervention?	Qual	VI	Prescribers felt engaged and were satisfied with the intervention. Pr 3: “*I definitely believe fewer pills are better.*” They also thought the recurrent attention and information improved PBM adherence. Pr 2: “*It is good that attention is paid [to this subject].”* Prescribers considered the intervention broadly applicable: Pr 1: “*99% of the patients use PBM, so I think it is deployable for everyone.*” The prescribers considered adequate communication and collaboration with prescribers and dietitians, essential aspects of the intervention. Pr 2: “*I was kept very much informed by mail.*”	
*Feasibility*					
General	How feasible is the intervention in daily practice?	Qual	IV, V, VI, VII	Screening should be improved. The PIDO-P intervention should be targeted at patients with an SPC > 2.0 mmol/L, using individualized target ranges. Recurring consultations could prove beneficial in keeping patients engaged over time. Adequate collaboration with and communication between involved healthcare professionals were seen as prerequisites. Pr 2: *“I think a good protocol is needed for that, that we know who does what and when, so to speak. And maybe also collaborate with the dietitians.*”	

I: study administration by the researchers; II: pharmacist perspective on pharmacist-patient consultations, oral survey; III: patient perspective on pharmacist-patient consultation, oral survey; IV: semi-structured interviews with pharmacists; V: semi-structured interviews with patients; VI: semi-structured interviews with prescribers; VII: electronic medical records

IM, intervention module; PBM, phosphate-binding medication; Ph, pharmacist; PIDO-P, pharmacist intervention and dose optimisation of phosphate-binding medication; Pr, prescriber; Pt, patient; QBS, quick barrier scan; RALPH, recognising and addressing limited pharmaceutical literacy; SPC, serum phosphate concentration; TIG, tailored intervention guide

### Feasibility

Barriers and facilitators were identified to guide the feasibility and implementation of the PIDO-P intervention in clinical practice. These barriers and facilitators were explored during semi-structured interviews with pharmacists, patients, and prescribers. To explore barriers and facilitators for implementation, questions were formulated based on earlier research using CFIF in pharmacist interventions [16, 17, 21] and adapted to the PIDO-P intervention and the haemodialysis context.

### Data collection

#### Data sources

From the start of the PIDO-P study in October 2021 until October 2023, data from research administration, oral surveys, and semi-structured interviews were used to assess IF, including adherence to the intervention and moderating factors. Feasibility was assessed using data from research administration and semi-structured interviews.

Research administration (data source I) contained qualitative and quantitative data on selection (number of patients screened, number of patients excluded), inclusion (how many patients were invited and how many patients agreed to participate), the number of consultations, drop­out, the logistics, and process of the performance and implementation of the intervention.

The oral surveys were performed directly after each of the first three consultations and regarded the perspectives of three pharmacists (data source II, quantitative data) and 59 patients (data source III, qualitative and quantitative data) on these consultations (see Supplementary Data 1 for the surveys). The surveys consisted of statements with answer options on a 3-point Likert scale (agree, neutral, disagree). For patients, the survey after the third consultation also contained one closed and four open-ended questions.

Semi-structured interviews were performed with two pharmacists (data source IV), six patients (data source V), and three prescribers (data source VI), and were used to qualitatively explore the IF of the PIDO-P intervention, its feasibility in clinical practice, and the respondents’ views on the intervention and the role of the pharmacist in the treatment with PBM. The semi-structured interviews were performed using a topic list per category respondent (pharmacist, prescriber, and patient, see Supplementary Data 2) and conducted by one of the participating pharmacists (SC). The topic lists were developed by the coordinating pharmacist (FJvdO).

Participant recruitment for the semi-structured interviews for pharmacists and prescribers was based on convenience sampling. Patients who participated in the semi-structured interviews were selected from the group of patients without difficulties with medication-related health literacy. Medication-related health literacy was assessed at baseline with the RALPH interview guide [22, 23]. Fourteen patients did not experience any difficulties with health literacy, of whom six participated in the semi-structured interviews. We chose this particular subset of the study population, intending to maximise the output of the semi-structured interviews, as most patients, and especially those with limited health literacy, did not provide elaborate answers in response to the open-ended questions from the oral surveys.

Data regarding patient characteristics and medication information were extracted from electronic medical records of the hospital information system Chipsoft Hix (version 6.2.3, Chipsoft, Amsterdam, the Netherlands) and the dialysis department system Diamant version 3.17.3 (Diasoft, Leusden, the Netherlands) (data source VII).

### Data analysis

Quantitative data were analysed using SPSS Statistics for Windows version 29.0.2 (IBM Corporation, Armonk, NY, USA). Categorical variables were reported as frequencies with percentages. Continuous variables were reported as means with standard deviation (SD) or medians with interquartile range (IQR), depending on the normality of distribution.

Qualitative data were analysed using Atlas.ti for Windows, (version 24, Scientific Software Development GMBH, Berlin, Germany). Semi-structured interviews were audio-recorded and transcribed verbatim. The transcripts were analysed using the thematic theory analysis according to Braun and Clarke [24]. Two independent researchers (FJvdO and SC) coded the transcripts based on the interview topic list. Subsequently, the coded transcripts were classified into broader themes. Differences were discussed until a consensus was reached. A third researcher (JGH) was involved if consensus was not reached.

### Ethics approval

The study was conducted according to the principles of the Declaration of Helsinki (October 2013). The study was assessed by the accredited Medical Ethics Committee (METC) of MEC-U (Medical Ethical Committee Utrecht) and deemed not to be subject to the Medical Research Involving Human Subjects Act (WMO), approval letter W21.142.

## Results

### Adherence to the intervention

Details in outcomes regarding the adherence to the intervention (coverage, content, frequency, and duration) are described in Table 1.

#### Coverage

Of the 369 patients screened for eligibility, 80 patients were potentially eligible and asked to participate; 75 patients provided informed consent. Participating pharmacists considered screening and assessing eligibility labour-intensive, as applying the inclusion and exclusion criteria was not automated. The screening process should be improved in the future to enhance the effectiveness of the intervention. The drop-out rate was relatively low and was exclusively caused by natural causes.

#### Content

The content of five out of six key components was delivered to a high degree. The written summary (part of key component E) was delivered to a moderate degree (in 65% of the patients), but patients and pharmacists regarded its usefulness as limited. In general, patients, pharmacists, and prescribers thought the PIDO-P intervention improved understanding and knowledge.

#### Frequency and duration

First consultations were carried out in 75 patients, second consultations in 73 patients, and follow-up (third) consultations in 72 patients. The total consultation time for three consultations was approximately 60 min. The number of days (mean ± SD) between consultation 1 and consultation 2 was 14.8 ± 8.3. The number of days between consultations 2 and 3 was 96.7 ± 29.8.

### Moderating factors

In Table 2, detailed outcomes regarding the moderating factors are described.

#### Intervention complexity

Pharmacists perceived the intervention description in the protocol and the intervention materials (QBS, RALPH, TIG) as clear and easy to use. With these materials, they were able to effectively discuss the selected IMs from the TIG with the patients. The first and second consultations did not contain any difficult aspects, whereas the description of the third consultation was not completely clear: pharmacists did not always understand what they needed to discuss with the patient during this consultation. Therefore, if necessary, pharmacists received extra information and training for this consultation.

#### Facilitation strategies

Several strategies were combined to support the implementation of the PIDO-P intervention for the participating pharmacists (individualized training, coaching, and support by the lead pharmacist (FJvdO)). The pharmacists perceived these facilitation strategies as helpful.

Before the start, the study and the PIDO-P intervention were presented to other involved HCPs (nephrologists, nurse practitioners, dietitians, and dialysis nurses). During the study, nephrologists, nurse practitioners, and dietitians received a monthly update. Notwithstanding these updates, the nephrologists found it hard to be aware of which patient actively participated in the study at a specific moment. All prescribers reported good collaboration with the researcher and coordination of treatment of high phosphate levels during the study.

#### Quality of delivery and participant responsiveness

In 59 patients, the intervention was evaluated after the third pharmacist-patient consultation. Patients were generally satisfied. They appreciated receiving information about phosphate and PBM. They also felt supported by the pharmacist to implement the use of PBM in their daily routine and to choose the most appropriate PBM according to their preferences.

During the semi-structured interviews, patients mentioned they had low expectations of pharmacists regarding their role in CKD patient care in general. Most of them only expected pharmacists to dispense the correct medication (patient 5: “*Dispensing the correct medication is the most important task* “) and to check for interactions (patient 4:”*Thinking about combinations of medications*”). One of the patients mentioned that she expected the pharmacist to provide accurate medication information (patient 1:”*The pharmacist, in my opinion, has more knowledge about [medication] and more experience*”).

During the semi-structured interview prescribers expressed their opinion on the pharmacist’s role in PBM treatment. Important aspects were providing information (prescriber 1:”*Explaining to patients about the correct timing [of PBM] and to provide dose adjustment advice*” and” *Informing patients about why patients have to take it during protein-rich meals*”), counselling (prescriber 3: “*Motivational interviewing and counselling*” and “*I think the pharmacist knows best [about PBM]*”), and recurrent attention to PBM (prescriber 2: *“Helping [patients] to take [PBM] correctly, and recurrent attention*”).

During the semi-structured interviews, HCPs mentioned that they believed that the intervention was broadly applicable. They also reported that incorporating patient preferences in an individualized treatment scheme, and adequate communication and collaboration between all HCPs involved were important aspects of the intervention. Furthermore, HCPs considered an adequate patient-HCP relationship necessary to optimize the benefits of the intervention, furthermore sufficient human and financial resources were considered essential.

### Mixed-methods integration: themes regarding the implementation fidelity of the PIDO-P intervention

After thematic analysis of the semi-structured interviews, data integration was performed using qualitative and quantitative data, generating metainferences. After data integration, four themes were established: (1) improving patient knowledge and understanding; (2) helping patients solve practical problems with PBM and improving the correct use of PBM; (3) improving shared decision-making and PBM treatment individualisation; (4) building the relationship between pharmacist and patient. Table 3 shows a joint display of these themes.

**Table 3:** Joint display of the four themes regarding the implementation fidelity of the PIDO-P intervention

	Quantitative results	Qualitative findings and quotes	Metainferences
Theme 1: Improving patient knowledge and understanding	The pharmacist thought the consultations improved understanding in 70% (30/43) of the patients at the end of the 1 st consultation and 79% (30/38) of the patients during the 2nd consultation 94% (34/36) of the patients reported better knowledge at the end of the 2nd consultation. 88% (52/59) of the patients reported at the end of the 3rd consultation that they found the consultations informative and useful	Ph 1: “*We should motivate patients and help them understand.*” Ph 2: “*Explain to patients, show them*.” Pt 1:”*The types of PBM, but also what it does exactly. A nephrologist and dietitian can also explain it, but in my opinion, the pharmacist has more experience with or knowledge of [medication].*” Pt2: “*I received a lot of information.*” Pr 1:”*It [the study] helps to focus on the tablets and the importance of taking them.*” Pr 2: “*It is also lifestyle and awareness.*”	The PIDO-P intervention promotes patient knowledge and understanding of PBM. Pharmacist consultations are of added value in addition to treatment by nephrologists, nurse practitioners, and dietitians
Theme 2: Helping patients solve practical problems with PBM and improving the correct use of PBM	88% (52/59) of the patients reported at the end of the 3rd consultation that they thought the consultations helped them to take PBM correctly	Pt 1: “*In the beginning, I had problems taking the medication during the day. I forgot to take them with me. Now, I take them with me in my bag.*“ and “*If I had problems taking them in the evening, then we would together search for a solution. I learnt to bring my tablets during the day.*” Pt 5:”*I’ve got the bottle [with PBM] beside the table. Then I see it and don’t forget [to take them]*. “	Addressing and, if possible, solving practical problems as barriers to patient adherence stimulates patient empowerment and self-management and involvement in shared decision-making. Furthermore, the PIDO-P intervention improves the correct use of PBM, as is shown by the results of the intervention on PBM pill burden (higher self-reported adherence with a reduction from 8.8 to 5.8 tablets while maintaining a stable SPC)
Theme 3: Improving shared decision-making and PBM treatment individualisation	94% (34/36) of the patients felt involved in the decision about dose adjustment and choice of phosphate-binding medication	Pt 4: “*We decided together to reduce the number of tablets.*” Pt 6: “*Then, we decided together to keep it at six pieces [tablets] a day.*”	Involving the patient in the dose reduction and asking about their PBM treatment preferences, while taking into account their dietary habits, stimulates shared decision-making and treatment individualisation
Theme 4: Building the relationship between pharmacist and patient	All patients felt listened to during the 1 st (n = 43) and 2nd (n = 36) consultation. All patients (n = 36) reported they had sufficient opportunity to ask questions	Patients valued the consultations, the clear communication, and the pharmacist’s attention. Patients and pharmacists would prefer recurrent visits by a single pharmacist. Pt 6: “*A familiar face would be nice.*” Ph 1: “*You develop another relationship. You can create a relationship faster with patients I already saw for the first consultation than with patients I only met at the second or third consultation.*”	Part of building the relationship between patient and pharmacist is creating trust. Patients seemed to readily trust the pharmacist, as they easily discussed barriers to adherence and the problems they encountered using PBM. Regular, recurrent, pharmacist-patient consultations, for example, every three months, by a single pharmacist, would probably increase the effectiveness of the intervention

### Feasibility and implementation in clinical practice

Combining data from the research administration, oral surveys and the semi-structured interviews with patients, pharmacists, and prescribers (data sources I, III–VI) led to the emergence of three important aspects of feasibility of the PIDO-P intervention.

1. Improving the intervention
  - Improvement of screening, as pharmacists considered this inefficient and only 20% of screened patients could be included (data source I and IV).
  - Recurrent follow-up consultations (every three months) by a single pharmacist (data source IV, V, VI)
  - Including patients with language barriers (data source IV and VI)
2. Targeting the intervention
  - Targeting the intervention at younger patients (data source VI)
  - Targeting the intervention at patients with higher serum phosphate concentration (> 1.8–2.0 mmol/L) (data source VI)
3. PBM treatment individualisation
  - Individualising target serum phosphate concentrations based on life expectancy and eligibility for transplantation (data source VI)
  - Individualising the choice of PBM, as patients generally have a preference for a specific dosage form: e.g. filmcoated tablets, chewing tablets, or powder (data source I and III)
  - Fitting PBM into daily life: taking into account dietary preferences, helping patients finding solutions to the practical problems they encounter in using PBM (data source I and III)

Prescribers considered both pharmacist-prescribing of PBM and recurring consultations regarding phosphate and PBM beneficial for the effectiveness of the intervention.

## Discussion

### Statement of key findings

This study aimed to evaluate the IF and feasibility of the multi-component, PIDO-P intervention and to identify moderating factors that might have influenced the implementation and performance of this intervention, as well as feasibility and barriers and facilitators for future implementation in clinical practice.

The results of this study show that the IF was moderate to high for all key components, with a lower score for coverage due to the inefficient screening and selection process. As all key components except for the written summary were implemented as intended, suboptimal IF of the PIDO-P intervention is unlikely to explain the absence of a short-term effect of this intervention on the SPC.

Four themes regarding the IF of the PIDO-P intervention could be identified: (1) improving patient knowledge and understanding; (2) helping patients solve practical problems with PBM and improving the correct use of PBM; (3) improving shared decision-making and PBM treatment individualisation; (4) building the relationship between pharmacist and patient.

Three important themes regarding feasibility and implementation in clinical practice emerged: the intervention should be improved, it should be targeted at specific patients, and PBM treatment should always be individualised.

An interesting discrepancy was observed when analysing the patient data from the evaluations and the semi-structured interviews. During the evaluations, performed immediately after the consultations, patients were positive about the intervention. They felt supported and thought the consultations helped them to correctly use phosphate-binding medication. However, during the semi-structured interviews, when they were asked about the possible role of the pharmacist in patients on haemodialysis, they generally only mentioned dispensing the correct medication. Two main factors may explain this discrepancy, the first being recall bias, as for some patients, the semi-structured interviews took place a considerable time after the consultations. Second, notwithstanding the active role of the pharmacist in the PIDO-P study, the pharmacist’s involvement in haemodialysis treatment is generally not visible to patients. All in all, the findings of the semi-structured patient interviews illustrate that haemodialysis patients have low expectations regarding the contribution of the pharmacist in their PBM treatment.

### Strengths and limitations

The first strength of this study is that we used a mixed-methods study design using several data sources, in which the qualitative aspects provided valuable insights into the thoughts and opinions of patients, pharmacists, and prescribers. The second strength is that we used a theoretical framework to evaluate IF, namely Carroll’s Conceptual Framework for Implementation Fidelity [20]. In literature, the use of a conceptual framework has been recommended to systematically evaluate the IF of an intervention [20, 25].

The first limitation is that we only interviewed a small number of HCPs and patients for the semi-structured interviews due to restricted availability. Furthermore, during these interviews, most patients shared a limited amount of information and, even when explicitly asked, were often not able to provide recommendations or suggestions for improvement. Therefore, the information density of data from the semi-structured patient interviews was very low, and data were probably not saturated. Another limitation is that certain aspects of the framework (e.g. quality of delivery) were assessed only to a limited extent. For example, we did not record pharmacist-patient consultations to analyse these consultations more profoundly. The last limitation is that the PIDO-P intervention, including the evaluation of its IF, was performed in only one hospital. This may have led to a more selective assessment of possible feasibility issues. However, the intervention was performed by trained pharmacists in a specialized hospital with a large haemodialysis unit, with intensive collaboration between prescribers, pharmacists, and other healthcare providers.

### Interpretation

Three other studies described the IF of multicomponent pharmacist intervention programs [17, 21, 26]. Similarities and differences exist between these studies and our study.

Regarding adherence to the intervention, in all four studies, the key intervention components were implemented to a moderate to high degree. However, differences in coverage and the delivery of complex key components (content) were seen. In our study, the coverage was higher (with exception of screening efficiency) and complex components were more adequately implemented. Two aspects of our study particularly facilitated patient participation (coverage): pharmacist-patient consultations were performed during haemodialysis sessions, and part of the intervention was a dose reduction of PBM, which was very appealing to patients. The complex components may have been more adequately implemented in our study due to the single-centre design, which facilitated intensive monitoring and training by the coordinating pharmacist. Both the CATI study [17] and our study implemented the written summary with only moderate fidelity. In both studies, the pharmacists considered a written summary of limited value, and patients rarely used it. This led the coordinating pharmacist in our study to discontinue providing a written summary in patient 62 to patient 75.

Regarding moderating factors, several similarities were observed: facilitation strategies were adequate in all four studies, with extensive pharmacist training and a structured protocol with a detailed intervention description. Furthermore, the quality of delivery was moderate to high in all four studies, and participants were generally responsive to and satisfied with the intervention. Differences in moderating factors were mostly seen in perceived intervention complexity, which was high in the MARCH (Medication Actions to Reduce hospital admissions through a collaboration between Community and Hospital pharmacists) study [21] and somewhat lower in the other studies. Several barriers to implementation in clinical practice (feasibility) were encountered in more studies: lack of human and financial resources were considered barriers to implementation in all four studies. Furthermore, an ineffective screening and selection process was considered a barrier to future implementation in our study, as well as Van der Laan’s [17]. In this study, prescribers considered targeting the PIDO-P intervention to patients with higher SPC as a way to improve its effectiveness. This suggestion is supported by a post-hoc analysis of data from the effectiveness part of the study: the intervention reduced SPC in patients with a baseline SPC > 2.0 mmol/L from 2.46 to 2.17 mmol/l (n = 29, *p* = 0.019) [data on file].

### Further research

To increase its effectiveness and feasibility, the PIDO-P intervention should be improved and targeted. Redesigning the patient screening and selection process, preferably by automatically combining laboratory and medication data, and targeting the intervention at patients with an SPC > 2.0 mmol/L, may significantly improve its effectiveness and feasibility in clinical practice. The effects of these changes in the PIDO-P intervention should be studied, if possible, also in a hybrid effectiveness implementation design, before wide-scale implementation in clinical practice can be recommended.

## Conclusion

The PIDO-P intervention was implemented with moderate to high fidelity and seems feasible in clinical practice, but the intervention needs further improvement and targeting. If these adjustments are proven successful, the PIDO-P intervention provides a good opportunity for a more active role of clinical pharmacists in PBM treatment.

## Supplementary Information

Below is the link to the electronic supplementary material.

Supplementary file1 (DOCX 31 KB)

Supplementary file2 (DOCX 25 KB)

## Data Availability

Data available on reasonable request.
