# Supplementary material for: Implementation fidelity of a pharmacist-led intervention program to improve a high serum phosphate concentration in haemodialysis patients: a mixed-methods study
Source: Int J Clin Pharm. 2025 Sep 26;48(2):446–68. doi: 10.1007/s11096-025-01995-z (PMC12992437; doi:10.1007/s11096-025-01995-z)
Supplement: Supplementary file 1 — Supplementary file1 (DOCX 31 KB) [file 11096_2025_1995_MOESM1_ESM.docx]

# Supplementary data 1. Oral surveys on the evaluation of pharmacist-patient consultations

The first three consultations were evaluated immediately after the consultations by performing an oral survey. The surveys consisted of several statements for patients and pharmacists, with answer options on a 3-point Likert scale (agree, neutral, disagree). Furthermore, after the survey, patients and pharmacists could provide remarks. After the third consultation, patients were asked to evaluate all previous consultations, including the third consultation.

## Consultation 1: Patient perspective on pharmacist-patient consultations: oral survey

### Statements

1. The consultation took place in a pleasant atmosphere
2. I felt like I was being listened to
3. I found it pleasant to talk about my phosphate binders
4. I understood all the questions that were asked
5. The duration of the consultation was appropriate

## Consultation 1: Pharmacist perspective on pharmacist-patient consultations: oral survey

### Statements

1. The patient understood all the questions that were asked
2. The consultation took place in a pleasant atmosphere
3. The consultation led to a better understanding by the patient
4. The consultation went as planned

## Consultation 2: Patient perspective on pharmacist-patient consultations: oral survey

### Statements

1. I now have more knowledge about phosphate and phosphate binders
2. I felt like I was being listened to
3. I had sufficient opportunity to ask questions
4. I was involved in the decision about dose adjustment and the choice of a phosphate binder
5. The duration of the consultation was appropriate

## Consultation 2: Pharmacist perspective on pharmacist-patient consultations: oral survey

### Statements

1. The consultation took place in a pleasant atmosphere
2. The consultation led to a better understanding by the patient
3. The consultation went as planned

## Consultation 3: Patient perspective on pharmacist-patient consultations: oral survey

### Statements

1. The summary I received on paper was helpful
2. I found the number of consultations sufficient
3. I found the consultations with the pharmacist about my phosphate and phosphate binders informative and useful
4. I would recommend participation to another patient
5. The information and advice I received helped me take the phosphate binders correctly

### Closed question

1. If you had to provide a score for the consultations with the pharmacist, what score would you give? (1–10)

### Open-ended questions

1. How did you find these consultations? Did they help you in using PBM? If so, how?
2. Do you have suggestions for the improvement of these consultations?
3. What are the advantages of these consultations?
4. What was your reason for participating in this study?
