# Supplementary material for: Implementation fidelity of a pharmacist-led intervention program to improve a high serum phosphate concentration in haemodialysis patients: a mixed-methods study
Source: Int J Clin Pharm. 2025 Sep 26;48(2):446–68. doi: 10.1007/s11096-025-01995-z (PMC12992437; doi:10.1007/s11096-025-01995-z)
Supplement: Supplementary file 2 — Supplementary file2 (DOCX 25 KB) [file 11096_2025_1995_MOESM2_ESM.docx]

# Semi-structured interviews

## Semi-structured interview with patients

I’m curious about your experience with the consultations about phosphate binders. These consultations took place during the period from ………………. to ………………… (Explanation of the intervention: a combination of consultations and dose reduction of phosphate binders). We would like to hear your opinion about these consultations.

1. Is there anything you would like to share about the consultations?
   (For example, were there any questions during the consultations that you found difficult? If so, which ones and why?)
2. What problems are you currently experiencing when taking phosphate binders?
3. How could the hospital pharmacist help you with taking phosphate binders?
4. What is your view on the role of the hospital pharmacist in the treatment of high phosphate levels?
5. What do you expect from a consultation with the hospital pharmacist about phosphate binders?
6. Do you have any tips for us (based on the consultations you had)?
7. Do you have any remaining questions?

## Semi-structured interview with pharmacists

1. What are your experiences with this study?
   - How involved and satisfied were you with the intervention (participant responsiveness)?
   - To what extent did you need help during this study?
   - What went well?
   - How involved were you with the intervention?
2. What feedback did you receive from the patients?
3. What points of improvement did patients mention during this study?
   - To what extent did patients need help during this study?
4. How do you experience the collaboration with the nephrologists within the context of this study?
5. What problems did you encounter during this study?
6. In what way (e.g., through training?) was the implementation of the consultations supported?
   - How did you experience the support/training (facilitation strategies)?
   - Did you miss anything in that regard — and if so, what?
7. Were you able to carry out the consultations based on the protocol description?
   - How detailed was the protocol description?
   - Would a summary-level description have been sufficient?
8. To what extent were you able to carry out the consultations as planned?
9. In your opinion, what should be the pharmacist's role in the treatment with phosphate binders?
   - What could the pharmacist’s role be, and what could we as pharmacists do better?
10. Suppose we want to implement this intervention as standard care — what do you think is necessary for that? Do you have any tips for the implementation?
11. Do you have any other tips regarding the intervention?

## Semi-structured interview prescribers

1. What are your experiences with this study?
2. What feedback do you receive from patients about the study?
3. How is the coordination with the pharmacy in the context of this study?
4. In your opinion, what is the added value of this intervention?
5. What should be the goal of the pharmacist’s involvement in the treatment with phosphate binders, in your view?
6. How well does the intervention align with existing workflows and practices in the hospital?
   Not / partially / mostly / completely
7. What could be improved in that regard?
8. Is the intervention broadly applicable? Is it suitable for all patients with poor phosphate control and high pill burden? If not, for whom is it not suitable? Do you think this applies to all dialysis centers in the Netherlands?
9. Are you encountering any problems in the treatment with phosphate binders during this study? If yes, which ones?
10. What could we, as pharmacists, do better?
11. Suppose we want to implement this intervention as standard care — what do you think is needed for that?Do you have any tips for the implementation?
12. Do you have any other tips regarding the intervention?
